# Amelioration by *Idesia polycarpa* Maxim. var. vestita Diels. of Oleic Acid-Induced Nonalcoholic Fatty Liver in HepG2 Cells through Antioxidant and Modulation of Lipid Metabolism

**DOI:** 10.1155/2020/1208726

**Published:** 2020-10-20

**Authors:** Na Li, Yi-ran Sun, Li-bo He, Lei Huang, Ting-ting Li, Tao-yu Wang, Lin Tang

**Affiliations:** ^1^Key Laboratory of Bio-Resources and Eco-Environment of Ministry of Education, College of Life Sciences, Sichuan University, Chengdu, 610065 Sichuan, China; ^2^Sichuan Testing Center for Biomaterials and Medical Devices, Chengdu, 610064 Sichuan, China; ^3^Institute of New Energy and Low-Carbon Technology, Sichuan University, Chengdu, 610065 Sichuan, China

## Abstract

*Idesia polycarpa* Maxim. var. vestita Diels (*I. polycarpa*) is well known as an edible oil plant which contains abundant linoleic acid and polyphenols. The objective of this study was to maximize the by-product of defatted fruit of *I. polycarpa*. We found that the fraction D of ethyl acetate extract (EF-D) contained more polyphenols, which contribute to its strong antioxidant activity by antioxidant assays (DPPH, ABTS, and FRAP). Meanwhile, EF-D showed a significant lipid-lowering effect on oleic acid- (OA-) induced hepatic steatosis in HepG2 cells through enhancing antioxidant activity, reducing liver damage, and regulating lipid metabolism, antioxidant, and inflammation-related gene expression. The SOD and T-AOC levels significantly increased, but the levels of MDA, AST, and ALT decreased obviously when treated with EF-D. In general, EF-D improved the antioxidant enzyme activities and decreased the hepatic injury activities. Besides, treatment with EF-D for NAFLD influenced lipid metabolism and inflammation by activating PPAR*α* which was associated with the increased expression of CPT1 and decreased expression of SCD, NF-*κ*B, and IL-1. Moreover, EF-D improved the oxidative stress system through activation of the Nrf2 antioxidant signal pathways and upregulated its target genes of HO-1, NQO1, and GSTA2. The results highlighted the EF-D from the defatted fruit of *I. polycarpa* regarding lipid-lowering, proving it to be a potential drug resource of natural products for treating the nonalcoholic fatty liver disease (NAFLD).

## 1. Introduction

Nonalcoholic fatty liver disease (NAFLD) encompassed a broad-spectrum pathology from simple triglyceride (TG) deposition in hepatocytes to nonalcoholic steatohepatitis (NASH), liver cirrhosis, and even hepatocellular carcinoma (HCC) [1]. With the change of life, a more calorie diet and less exercise have caused a rise among the general population in recent years [2]. It has been proven that oxidative stress involved in the etiology of NASH caused an imbalance between prooxidant and antioxidant chemical species that led to oxidative damage of cellular macromolecules [3–7].

Related reports showed that the damage of the membrane system will lead to the increased oxygen pressure in NAFLD and can initiate an oxidative stress response. Furthermore, multiple metabolic pathways of NAFLD can be mediated by PPARs to regulate lipid metabolism, glucose metabolism, and immune pathways. At present, there is no specific drug for the effective treatment of NAFLD due to its unclear pathogenesis and “crosstalk” between multiple metabolic pathways [8, 9]. Based on the understanding of the current situation of NAFLD, it is urgent to develop new drugs and explore new therapeutic ways.

*Idesia polycarpa* Maxim. var. vestita Diels (*I. polycarpa*), a member of the Salicaceae family and the only species of monotypic genus Idesia, is a common oil woody and edible plant distributed widely in East Asia, such as China, Japan, and Korea. Research concerning the use of *I. polycarpa* as a natural source of edible oil and dietary supplement increased gradually due to its various unsaturated fatty acids, such as linoleic acid and linolenic acid [10, 11]. The relative report has been confirmed that the oil of its fruits is nontoxic and has anticarcinogenic and antidiabetic effects and antihypertensive properties [12, 13]. Moreover, flavonoid and phenolic glycosides of *I. polycarpa* exhibited various biological activities, such as antioxidant, skin whitening, inhibition of platelet aggregation, and antiadipogenesis [14]. Previous researches demonstrated that the ethyl acetate extracts of *I. polycarpa* exhibited strong antioxidant [14], anti-inflammatory, and whitening activities *in vitro* and *in vivo* [15, 16].

Taken together, these researches showed that *I. polycarpa* could be regarded as having potential medicinal value as a natural product for its antioxidant and anti-inflammatory activity. In our study, we isolated the EF-D from the defatted fruit of *I. polycarpa* by high-speed counter-current chromatography (HSCCC). Besides, we investigated the treatment effect and mechanism of EF-D-alleviated OA-induced NAFLD in HepG2 cells by antioxidant activities, related enzyme activities, and related gene expression. Our research has achieved the utilization by-product of the defatted fruit of *I. polycarpa* and demonstrated its potential medical value for alleviating NAFLD.

## 2. Materials and Methods

### 2.1. Reagents

Analytical-grade methanol, ethanol, acetonitrile chloride hexahydrate, ferrous sulfate, sodium molybdate, sodium nitrite, sodium hydroxide, sodium carbonate, Folin-Ciocalteu reagent (FC), and DPPH (Swell Scientific Instruments Co. Ltd., Chengdu, China). Cell Counting Kit-8 (CCK-8) was purchased from KeyGen Biotech (Jiangsu, China). Test kits for triglyceride (TG), aspartate aminotransferase (AST), alanine aminotransferase (ALT), malondialdehyde (MDA), superoxide dismutase (SOD), and total antioxidant capacity (T-AOC) were purchased from Nanjing Jiancheng Bioengineering Institute (Nanjing, China). BCA Protein Quantification kit was purchased from Thermo Fisher Scientific (Thermo, USA). Oleic acid (OA) and oil red O (ORO) were purchased from Sigma Chemicals Co. (St. Louis, MO). TRIzol reagent and SYBR Premix Ex Taq were obtained from Invitrogen and Takara, respectively.

### 2.2. Plant Material Preparation of Extracts

Fruits of *Idesia polycarpa* Maxim. var. vestita Diels (*I. polycarpa*, voucher specimen No. SZ 0071614) were collected from the local market (Guangyuan, Sichuan, China) and were identified by Jie Bai, School of Life Sciences, Sichuan University.

After being placed in the shade to dry up at a constant weight at room temperature, the fruits were ground and sieved with a 40-mesh sieve. Before analysis, the oil of samples was removed by n-hexane extraction 3 times and the solution was pooled. After vacuum filtration, the filtrate was combined and dried under vacuum at 37°C. The defatted powder was stockpiled at -20°C. Approximately 100 g of the defatted powder was weighed and extracted with 75% ethanol (1 : 40 g/mL) at 65-75°C for 4 h. The extracts were filtered, and the filtrates were concentrated by using a vacuum rotary evaporator. Before use, the primary 75% ethanol extract (EE) was suspended in distilled water. The solutions were sequentially extracted by ethyl acetate for 4 times. Lastly, the ethyl acetate extracts (EAE) were evaporated to dryness at 50°C.

### 2.3. Ultrahigh Performance Liquid Chromatography (UPLC) Analysis

The compounds present in EE and EAE were carried out on a Waters ACQUITY System focused instrument equipped with a binary high pressure pump, a photodiode array detector, a thermostatic column compartment, and an automated sample injector (Waters, Inc., America). Chromatographic runs were all performed using a reverse-phase column (C18, 250 × 4.6 mm, 5 *μ*m particle size, Phenomenex, America). The mobile phase consisted of solvent A—acetonitrile, and solvent B—0.1% aqueous formic acid solution; the flow rate was 0.5 mL/min and the column temperature was maintained at 40°C during the run. The elution program was 10%-25% A (0-1.0 min), 25%-30% A (1.0-2.0 min), 30%-31% A (2.0-2.4 min), 31%-32.5% A (2.4-4.4 min), 32.5%-35% A (4.4-5.5 min), 35%-10% A (5.5-5.51 min), and 10% A (5.51-8.0 min). Detection was performed at 280 nm. The injection volume was 10 *μ*L. All analyses were performed in triplicate.

### 2.4. High-Speed Counter-Current Chromatography (HSCCC) Separation of EAE

The EAE were separated by HSCCC. The peak fractions were collected manually according to the elution profile and evaporated under reduced pressure. Optimal two-phase solvent systems are evaluated with a partition coefficient (0.2 ≤ *K* ≤ 5). The two-phase solvent systems ethyl acetate‐n‐butyl alcohol‐water = 4 : 1 : 5, 2 : 3 : 5, and 3 : 2 : 5 and n‐hexane‐ethyl acetate‐methanol‐water = 2 : 5 : 2 : 5, 3 : 2 : 1 : 5, 3 : 5 : 3 : 5, and 4 : 1 : 1 : 5, *v*/*v*/*v*/*v*, were screened as follows: the two-phase solvent system was prepared by adding each solvent to a separatory funnel. After shaking and thoroughly equilibrating at room temperature, the upper phase and lower phase were then separated and degassed ultrasonically for 30 min. The upper phase was used as the stationary phase while the lower phase as the mobile phase. The sample solution for HSCCC separation was prepared by dissolving 5 g of EAE in 20 mL of the lower phase. The multilayer coil column was entirely filled with the stationary phase at a flow rate of 30.0 mL/min. The apparatus was rotated at 800 rpm, then the mobile phase was pumped into the column at a flow rate of 4 mL/min in the head to-tail elution mode. The mobile phase eluting at the tail outlet indicated that hydrodynamic equilibrium had been reached. Subsequently, 20 mL of sample solution was injected into the column. The detection wavelength was set at 280 nm.

### 2.5. Antioxidant Activities In Vitro

#### 2.5.1. Total Flavonoid (TFC) and Total Phenolic Content (TPC)

The method used to determine total phenolic content was based on the TFC method reported elsewhere with proper modification. Briefly, 10 *μ*L extracts were mixed with 100 *μ*L FC reagent, and 90 *μ*L 10% sodium carbonate solution was added 5 min later. The mixture was incubated at 25°C for 40 min with constant oscillation. The observance was gauged at a 765 nm wavelength. Gallic acid was used as a standard (0.002-0.025 mg/mL). The content of phenolics was calculated from a regression equation (*y* = 3.381*x* + 0.038, *R*^2^ = 0.9995) and expressed as mg gallic acid equivalent/g of defatted fruit (mg GAE/g). The total flavonoid content was determined based on a colorimetric method with aluminium chloride [17, 18]. Briefly, the 20 *μ*L extracts (0.1 mg/mL) or rutin (0-0.1 mg/mL) was diluted in 60% ethanol solution and mixed with 30 *μ*L of NaNO_2_ (5%). After 6 min, 50 *μ*L of 10% AlCl_3_ was added to the above mixture; subsequently, 100 *μ*L of NaOH (1 M) was added. After 15 min, the absorbance values were measured at 510 nm and compared with 50% ethanol as a blank control. The total flavonoid content was calculated from a regression equation (*y* = 0.4196*x* + 0.002, *R*^2^ = 0.9995) and expressed as rutin equivalents (RE) per g of dry extract.

#### 2.5.2. DPPH Radical Scavenging Assay

100 *μ*L of the extracts at different concentrations (6.25-150 *μ*g/mL, dissolved in 60% ethanol) was mixed with 100 *μ*L of DPPH solution (0.1 mM, in 60% ethanol). VC at the same concentrations was mixed with 100 *μ*L of DPPH solution (0.1 mM, in 60% ethanol). The mixed solution was allowed to stand for 30 min in the dark at 23-25°C, after which the absorbance was measured at 517 nm, with 60% ethanol as a blank control [19, 20].

#### 2.5.3. ABTS Radical Scavenging

The stock solutions included 7.4 mM ABTS+ solution and 2.6 mM potassium persulfate solution. The working solution was then prepared by mixing the two stock solutions in equal quantities and allowing them to react for 12 h at room temperature in the dark. The solution was then diluted by PBS to obtain an absorbance of 0.7 ± 0.02 units at 734 nm using the microplate reader. 100 *μ*L of various concentrations (6.25-120 *μ*g/mL, dissolved in ethanol) of the extracts was mixed with 100 *μ*L of the diluted ABTS + solution. The reaction mixture was incubated at 30°C for 30 min [21]. Then, the absorbance was taken at 734 nm using the microplate reader (SpectraMax M2, Molecular Devices, Sunnyvale, CA, USA). VC was used as a reference.

#### 2.5.4. FRAP Assay

25 *μ*L of different concentration extract solutions (dissolved in 60% ethanol) was mixed with 50 *μ*L of phosphate buffer saline (0.2 M, pH 6.6) and 25 *μ*L of 1% (*w*/*v*) K_3_Fe(CN)_6_ solution. After incubation at 50°C for 30 min, 50 *μ*L of 10% trichloroacetic acid (TCA) was added. Then, the upper layer was combined with a 60 *μ*L 0.1% (*w*/*v*) FeCl_3_ solution. The absorbance was analyzed at 700 nm (BHT was used as a positive control) [22–24]. Increased absorbance of the reaction mixture indicates a greater reducing power.

### 2.6. Cell Culture

Human hepatocellular carcinoma (HepG2) cells, obtained from American Type Culture Collection (HB-8065, VA, USA), were cultured in DMEM contained with 10% fetal bovine serum (FBS) (Gibco, USA) and 1% antibiotic (penicillin-streptomycin) (Gibco, USA) and incubated in a humid incubator at 37°Cand 5% CO_2_. When reaching approximately 80% confluency, the cells were treated with 1 mM oleic acid bovine (OA-BSA) complex (molar ratio 4 : 1) for 24 h, then treated with different concentrations of EF-D for 24 h.

### 2.7. Cell Viability Assay

1 × 10^4^ HepG2 cells/well were seeded in a 96-well plate for 24 h, then treated with the EF-D groups: cells were treated with 10, 20, 40, 60, 80, 100, 150, 200, and 400 *μ*g/mL of EF-D (dissolved by DMSO), and the OA+EF-D groups: cells were treated with (1 mM) OA followed by different concentrations of EF-D. Then, 10 *μ*L of Cell Counting Kit-8 assay (CCK-8) was added to each well and incubated at 37°C for 1 h. Lastly, absorbance readings at 450 nm were obtained using a microplate reader.

### 2.8. Measurement of Protein Concentration

The cell lysate proteins were quantified by using a BCA protein assay kit. Briefly, after treatment with the drugs, the cells were harvested and washed twice by centrifugation (Centrifuge 5424 R, Eppendorf, Germany) with precooled PBS at 600 × g for 5 min. The supernatant was discarded, and the cells were resuspended in 200 *μ*L of cell lysis buffer and incubated on ice for 10 min. After the second centrifugation (8000 × g, 5 min), the supernatants were transferred to a new centrifuge tube and placed on ice during protein level analysis using the BCA Protein Quantification Kit. The absorbance values were measured at 562 nm by using a microplate reader. The protein concentration was calculated from a regression equation (*y* = 0.0303*x* + 0.0997, *R*^2^ = 0.9877) and expressed as mg/mL.

### 2.9. Determination of Lipid Accumulation

#### 2.9.1. Measurement of Cellular Triglyceride (TG)

Cells were collected (less than 6 × 10^6^ cells) and mixed with 1 mL lysis buffer (2 : 1, *v*/*v*). The resulting mixture was shaken at room temperature (RT) for 20 min and then centrifuged (500 rpm) for 10 min. The bottom layer was collected and resuspended for analysis of TG. TGs were measured using an enzymatic method kit following the instructions of the manufacturer.

#### 2.9.2. Qualitative and Quantitative Measurements of Steatosis

The qualitative measurement of lipid accumulation was stained by using ORO. Cells were washed with PBS three times and fixed in 4% paraformaldehyde at room temperature for 40 min. Then, remove fixative and wash three times with PBS and stain with ORO for 30 min at RT. Removing the ORO solution, cells were washed with PBS three times, then were observed under a fluorescent inverted microscope [25, 26]. Then, adding isopropanol to split them, absorbance reading at 492 nm was obtained using a microplate reader.

### 2.10. Key Enzyme Measurement of Hepatic Function and Antioxidant Activities

#### 2.10.1. Determination of AST and ALT Activities

AST and ALT activities were determined by a commercial test kit regarding the manufacturer's instructions. The absorbance values were measured at 340 nm by using a microplate reader, and the activities of AST and ALT were expressed as U/L.

#### 2.10.2. Measurement of MDA

The content of MDA from cell lysis was related to lipid peroxidation which was measured by a commercial test kit. The absorbance values were measured at 532 nm by using a microplate reader and the content of MDA expressed as nmol/mg prot.

#### 2.10.3. Measurement of SOD

SOD activity was measured by using a commercial test kit with reference to the manufacturer's instructions. The absorbance values were measured at 450 nm by using a microplate reader and the activity of SOD was expressed as U/mg prot.

#### 2.10.4. Measurement of T-AOC

T-AOC was measured by using a commercial test kit concerning the manufacturer's instructions. The absorbance values were measured at 593 nm by using a microplate reader and T-AOC was expressed as *μ*M/mg prot.

### 2.11. Reverse Transcriptase Polymerase Chain Reaction (RT-PCR)

The total RNA of cells was isolated using a TRIzol lysis reagent (Qiagen Sciences, Germantown, MD), according to the instructions of the manufacturer. The mRNA was reverse-transcribed into cDNA with a reverse-transcription kit (Takara Bio Inc), employing a SuperScript One-Step RT-PCR kit (Invitrogen, Carlsbad, CA). RT-PCR products were run with a loading star (Dyne Bio, South Korea) on a 1% agarose gel. Relative expression levels were calculated using the 2^-*ΔΔ*Ct^ method and were normalized to GAPDH levels. The primer sequences are listed in Table 1.

### 2.12. Statistical Analysis

The data were expressed as means ± standard deviation (SD) of three replications per experiment. Analysis of variance (ANOVA) (SPSS15.0, SPSS Inc., Chicago, IL, USA) and GraphPad Prism5 software (GraphPad Software, USA) were used. Values of *p* < 0.05, *p* < 0.01 or *p* < 0.001 were considered statistically significant.

## 3. Results

### 3.1. Selection of Active Parts

**Figure 1:**
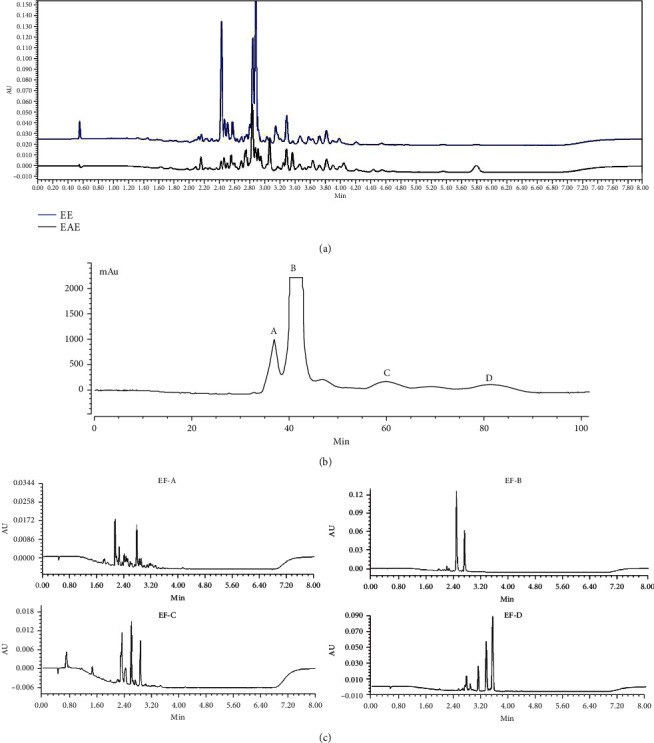
Chemical composition analysis: (a) UPLC chromatograms of EE and EAE detected at 280 nm; (b) four fractions of EAE isolated by HSCCC; (c) UPLC chromatograms of EF-A, EF-B, EF-C, and EF-D detected at 280 nm.

The compounds of EE and EAE from the defatted fruit of *I. polycarpa* were detected by UPLC (Figure 1(a)). We found that the activities of EAE were relatively stronger compared with EE which was consistent with previous reports. Base on this analysis, we establish a valid way to isolate and purify EAE for its utilization.

Our study confirmed the two-phase solvent system composed of n-hexane-ethyl acetate-methanol-water (3 : 2 : 1 : 5, *v*/*v*/*v*/*v*) as optimal for its appropriate *K* values and the retention of the stationary phase (43.7%). In the HSCCC separation, the fractions were separated in 100 min with a total mobile elution volume of 300 mL (Figure 1(b)). Four fractions were collected and detected by UPLC (Figure 1(c)). Fractions A of ethyl acetate extract (EF-A) and C (EF-C) were low in content and complex in composition. Therefore, we selected fractions B of ethyl acetate extract (EF-B), D (EF-D), EE, and EAE for further antioxidant assays.

**Figure 2:**
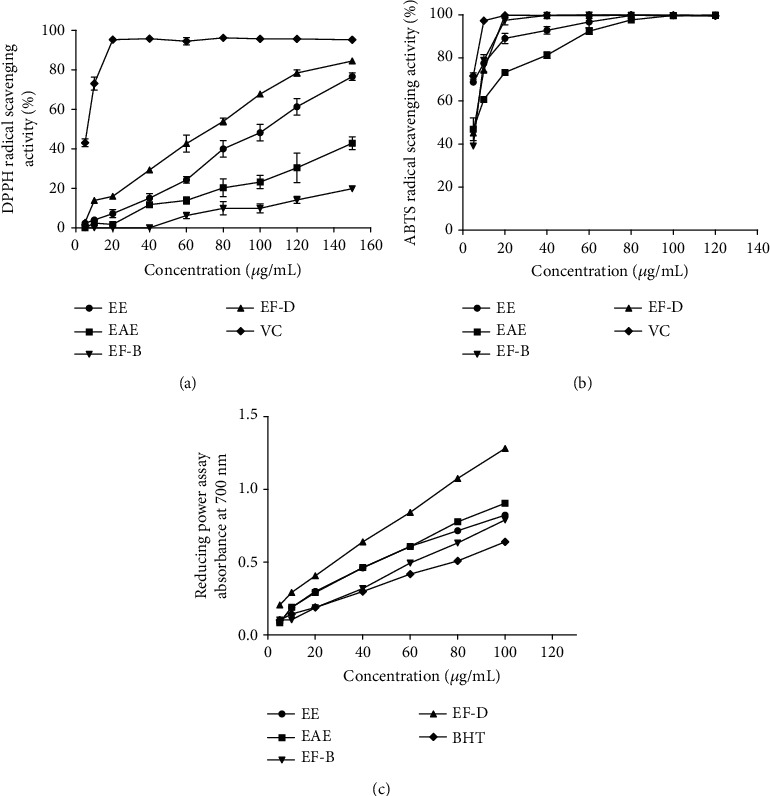
Effects of the antioxidant activities of the EE, EAE, EF-B, and EF-D: (a) DPPH assay; (b) ABTS assay; (c) FRAP assay. Data are presented as *means* ± *SD* (*n* = 3). VC: vitamin C; BHT: butylated hydroxytoluene.

The TPC and TFC of four different extracts are shown in Table 2. Among the four different extracts, EF-D showed the highest total flavonoid content, followed by EAE, EE, and EF-B. The phenolic contents of EE and EF-D are higher than others. In the DPPH scavenging assay (Figure 2(a)), the IC_50_ value (Table 2) (the concentration required scavenge 50% of radical) showed EF-D was the strongest activity than others. In the ABTS assay (Figure 2(b)), the activity was dose-dependent in low concentrations, and the IC_50_ value was VC > EE > EF − D > EF − B > EAE. FRAP was associated with the reduction of ferric ions to ferrous ions. At the concentrations of 5-100 *μ*g/mL, the phenomenon of concentration dependence was obvious, and EF-D showed the highlight activity by evaluating the value of *A*_0.5_ (Figure 2(c)). Comprehensively, EF-D was the strongest fraction in antioxidant activity compared to others and then was selected for lipid-lowering study *in vitro*.

### 3.2. OA Induced Hepatic Steatosis and Cell Viability

**Figure 3:**
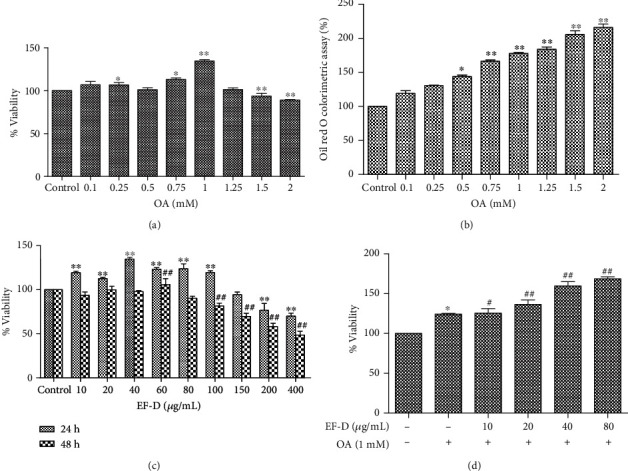
Cell viability and the TG accumulation effects of OA and EF-D:(a) HepG2 cells were treated with OA in different concentrations (0.1-2 mM) for 24 h; (b) cells were stained with ORO; (c) cell viability of EF-D for 24 h and 48 h; (d) cell viability of OA+EF-D. ^∗^*p* < 0.05 and ^∗∗^*p* < 0.01 versus control. ^#^*p* < 0.05 and ^##^*p* < 0.01 versus OA.

The HepG2 cells were treated with 0.1-2 mM concentration of OA for 24 h to induce hepatic steatosis. Compared to the control, it did not cause cytotoxicity to the cells when treated with 0.1-2 mM OA (Figure 3(a)). Cell viability reached a peak when treated with 1 mM OA. The number of lipid droplets in HepG2 cells was increased with the concentrations of OA (Figure 3(b)). Comprehensively, 1 mM OA was selected as a final concentration for inducing accumulation of lipid.

No evidence of toxicity of EF-D (10-200 *μ*g/mL) was found in the HepG2 cells after treating for 24 h. Cell toxicity of EF-D (100-400 *μ*g/mL) was obvious when treated for 48 h (Figure 3(c)). In addition, the viability was higher than the cells that were not treated with EF-D in a concentration-dependent manner (Figure 3(d)). Taken together, 10, 20, 40, and 80 *μ*g/mL of EF-D were chosen for further study.

### 3.3. Effects of EF-D on TG Accumulation in OA-Induced Hepatic Steatosis

**Figure 4:**
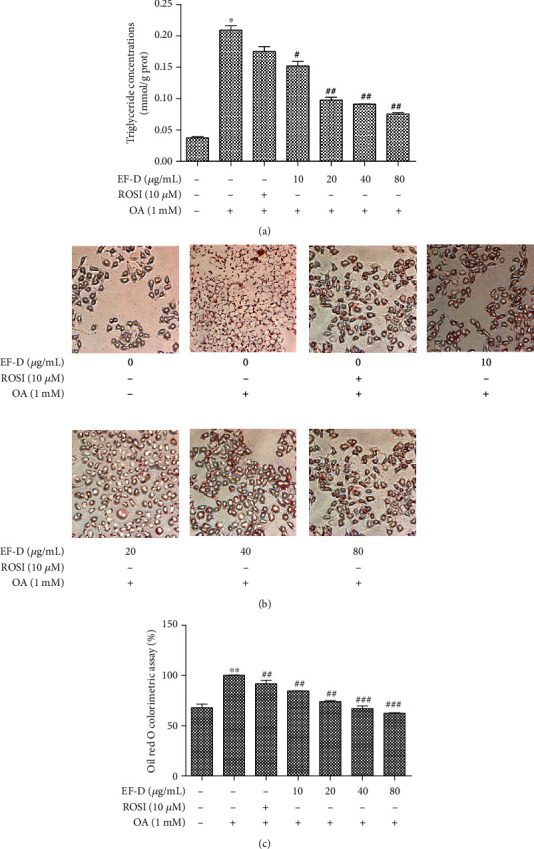
Lipid-lowering effect of EF-D: (a) total intracellular TG content; **(**b, c) cells were stained with ORO and then quantitatively analyzed. Data are represented as *mean* ± *SD* (*n* = 3). ^∗^*p* < 0.05 and ^∗∗^*p* < 0.01 OA versus control. ^#^*p* < 0.05, ^##^*p* < 0.01, and ^###^*p* < 0.001 OA+ROSI (10 *μ*M), OA+EF-D versus OA.

To examine the lipid-lowering role of EF-D, we measured the TG accumulation in HepG2 cells. TG accumulation was significantly increased in the OA group compared with the control group, while EF-D inhibited TG accumulation in a concentration-dependent manner when compared with the OA group. Rosiglitazone (ROSI) was recognized as the most effective and widely used medicine for NAFLD at present [27]. The inhibition of the lipid accumulation effect of EF-D was more sensitive to ROSI (Figure 4(a)). As shown in Figures 4(b) and 4(c), OA increased the intracellular lipid content when compared to the control. The number and size of lipid droplets were significantly reduced when EF-D was added compared with the OA group. Additionally, lipid deposits decreased by 10.2, 17.5, 24.7, 38.7, and 42.5% with 10 *μ*M (3.57 *μ*g/mL) ROSI and 10, 20, 40, and 80 *μ*g/mL EF-D treatments. These data initially implied that EF-D had a regulatory lipid-lowing effect on OA-induced hepatic steatosis.

### 3.4. Effects of EF-D on Key Markers in Responses to Oxidative Stress

**Figure 5:**
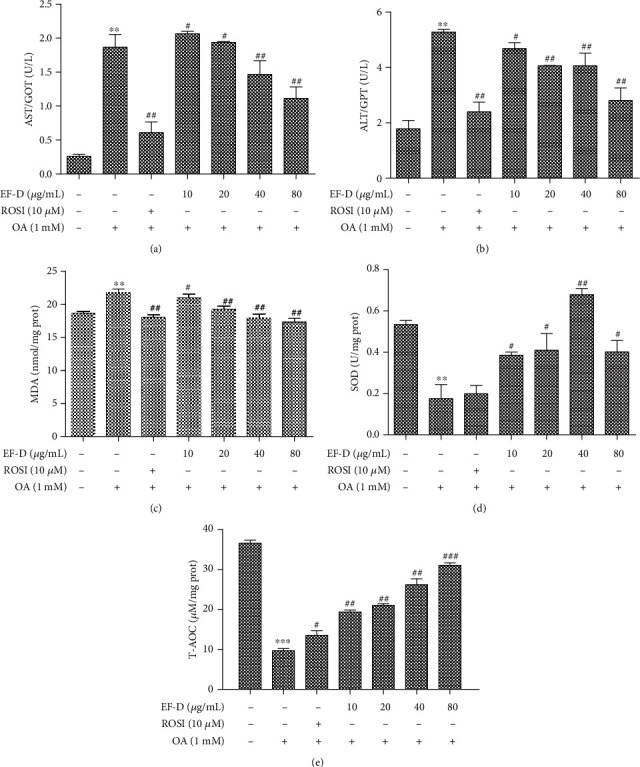
Effects of EF-D on key marker responses to oxidative stress: **(**a, b) AST and ALT activities of cellular supernatant; (c) the content of MDA in the supernatants of HepG2 cells lysates; (d, e) SOD and T-AOC activities in the supernatants of HepG2 cell lysates. Data are represented as *mean* ± *SD* (*n* = 3). ^∗∗^*p* < 0.01 and ^∗∗∗^*p* < 0.001 OA versus control. ^#^*p* < 0.05, ^##^*p* < 0.01, and ^###^*p* < 0.001 OA+ROSI (10 *μ*M), OA+EF-D versus OA.

In this study, the activities of AST and ALT in the OA-induced HepG2 cells were higher than those of the corresponding control group, while treatment with EF-D observably suppressed the increase of ALT and AST in a concentration-dependent manner compared with the OA group (Figures 5(a) and 5(b)). The results demonstrated that EF-D reduced lipid accumulation by lowering liver injury-related enzyme activities. MDA content is commonly known as a marker of oxidative stress, and its level can directly influence the peroxidation of membrane. The levels of SOD and T-AOC can reflect the capacity of the antioxidant. As the results showed, EF-D dose-dependently decreased the content of MDA (Figure 5(c)) and elevated the levels of SOD (Figure 5(d)) and T-AOC (Figure 5(e)) in comparison to the OA group which further confirmed that EF-D alleviated lipid accumulation by decreasing enzyme activities associated with liver injury and enhancing antioxidase activities.

### 3.5. The Effects of EF-D on the Expression of Antioxidant Genes Related to Hepatic Steatosis

**Figure 6:**
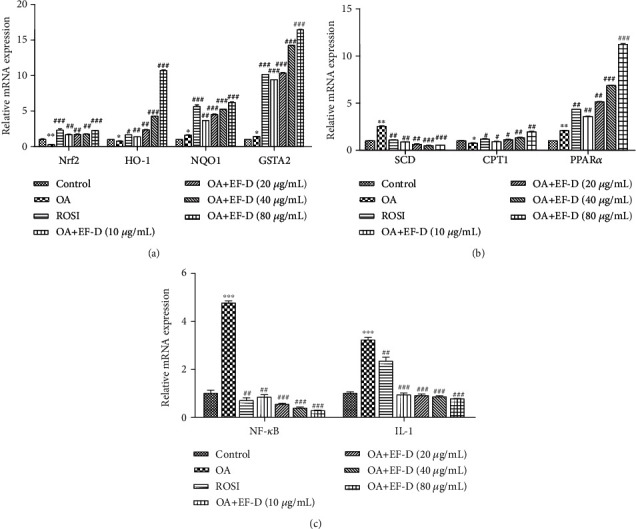
Effects of EF-D on the mRNA expression of oxidative stress and inflammatory cytokine genes: (a) Nrf2, HO-1, NQO1, and GSTA2; (b) SCD, CPT1, and PPAR*α*; (c) NF-*κ*B and IL-1 mRNA expression. Data are represented as *mean* ± *SD* (*n* = 3). ^∗^*p* < 0.05 and ^∗∗^*p* < 0.01 OA versus control. ^##^*p* < 0.01 and ^###^*p* < 0.001 OA+ROSI and OA+EF-D versus OA.

Nuclear factor erythroid-2-related factor 2 (Nrf2), a key transcription factor in oxidative stress, can activate the expression of downstream related genes [28]. Our results demonstrated that EF-D attenuated lipid accumulation through increasing mRNA expression of Nrf2 and upregulating mRNA levels of its downstream oxidative response genes including heme oxygenase-1 (HO-1), NAD(P)H dehydrogenase, quinone 1 (NQO1), and glutathione S-transferase alpha 2 (GSTA2) (Figure 6(a)).

### 3.6. The Effects of EF-D on the Expression of Lipogenic Genes and Inflammatory Cytokine Genes Related to Hepatic Steatosis

Stearoyl coenzyme A desaturase (SCD) and carnitine palmitoyltransferase-1 (CPT1) are key genes for the lipid synthesis pathway and lipid catabolism pathway. Our results showed that EF-D downregulated SCD and upregulated CPT1 mRNA expression when compared with the OA group. Peroxisomal proliferator-activated receptor *α* (PPAR*α*) is closely related to several important metabolic pathways. Our results showed that EF-D upregulated the mRNA expression of PPAR*α* which indicated EF-D regulated lipid metabolism by activating PPAR*α*. (Figure 6(b)).

Nuclear factor kappa-B (NF-*κ*B), tumor necrosis factor (TNF), and interleukin (IL) are the most critical factors associated with inflammation in NAFLD [29, 30]. These findings indicated that EF-D distinctly downregulated the mRNA expression of NF-*κ*B and IL-1 compared with the OA group (Figure 6(c)). These results implied that EF-D may play a potential role in preventing the process of inflammatory cytokines.

## 4. Discussion

**Figure 7:**
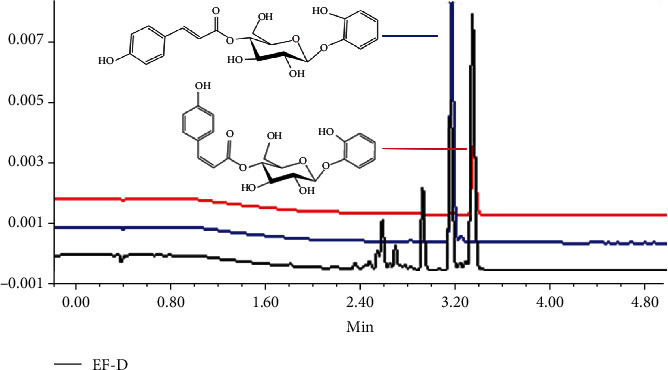
UPLC chromatograms of EF-D and two monomers detected at 280 nm.

In the recent years, multiple natural phytochemicals, including polyphenols and carotenoid components from food, have been demonstrated to behave with beneficial effects on suppressing the development of NAFLD through improving hepatic glycolipid metabolism dysfunction [31, 32]. However, there are not many reports on treating NAFLD with natural products. The purpose of the study was to explore the antioxidant and lipid-lowering effect of *I. polycarpa in vitro*. It has been reported that many natural products that are rich in polyphenols and glycosides can repress lipid accumulation [33, 34]. Our study found that the fragment EF-D from the defatted fruit of *I. polycarpa* can effectively ameliorate OA-induced NAFLD in HepG2 cells. Interestingly, we found that EF-D mainly contained 1-[(4′-O-(Z)-p-coumaroyl)-*β*-D-glucopyranosyl]-oxy-2-phenol and 1-[(4′-O-(E)-p-coumaroyl)-*β*-D-glucopyranosyl]-oxy-2-phenol [35] (Figure 7). We identified the two compounds by comparing to the standard substances which were isolated from the defatted fruit of *I. polycarpa* and identified by mass spectrometry in our laboratory [36]. The antioxidant activities and cell cytotoxicity of two new monomers have been proven [36]. Our findings are consistent with the previous studies and demonstrate that the defatted fruit of *I. polycarpa* acts as a natural product for ameliorating NAFLD.

Oxidative stress plays a key role in causing the NAFLD process. High activity molecules such as reactive oxygen (ROS) and reactive nitrogen (RNS) free radicals caused an imbalance between prooxidant and antioxidant chemical species that led to oxidative damage of cellular macromolecules [37, 38]. A previous study has reported that polyphenols can reduce the production of ROS and RNS [39]. The MDA, SOD, and T-AOC are important enzymes of the endogenous antioxidant defense system. Our results showed that EF-D decreases the level of MDA and increases the activities for SOD and T-AOC. Thus, the antioxidant capacity of EF-D seems to have an important connection with treating NAFLD. It has been reported that polyphenols through activation of Nrf2-ARE signal pathways respond to oxidative stress for inhibiting lipid accumulation [40]. Furthermore, Nrf2 promotes the activation of downstream relative target genes, such as HO-1, NQO1, CAT, and GSTA2, leading to improved oxidative stress resistance [41]. The expression of genes can influence the generation of relative protease which further regulated related metabolic pathways. It has been demonstrated that genetic control of all characters is mediated through specific enzymes. More precisely, the message of the gene is ultimately carried out by the enzymes [42]. Our results indicated that EF-D could decrease OA-induced oxidative damage through activation of the Nrf2 signaling pathway.

It has been reported that SCD can regulate lipid metabolism and further affect insulin sensitivity [43]. CPT1 is a key regulatory enzyme and rate-limiting enzyme for the beta-oxidation of long-chain fatty acids in liver tissues and cells, which is closely related to the catabolism pathway of fatty acids. Earlier studies showed PPARs were target genes for remission in multiple pathways that are disrupted in NAFLD [44]. In particular, PPAR*α* is located primarily in the liver, adipose tissue, kidney, heart, skeletal muscle, and large intestine where it is thought to regulate the fatty acid synthesis and oxidation, gluconeogenesis, ketogenesis, and lipoprotein assembly [45]. Besides, studies have demonstrated that inflammatory metabolism is closely related to oxidative injury and lipid metabolism [46]. In our experiments, EF-D decreased the mRNA expression of SCD, NF-*κ*B, and IL-1 and increased the mRNA expression of CPT1 and PPAR*α*. Taking the above results together, EF-D inhibited inflammation and improved lipid metabolism disorders through activating PPAR*α* and Nrf2 antioxidant pathways.

## 5. Conclusion

In conclusion, EF-D can improve the disturbance of lipid metabolism and inflammation in OA-induced hepatic steatosis in HepG2 cells through strong antioxidant activity. Moreover, the mechanism was achieved through regulating relative metabolic enzyme activities and activating PPAR*α* and Nrf2 antioxidant signal pathways. Evidences collected in this research suggested that EF-D is beneficial for lipid-lowering and acts as a candidate medicine for ameliorating NAFLD.

## Figures and Tables

**Table 1:** Sequences of primers used in RT-qPCR.

Gene	Forward primer (5′-3′)	Reverse primer (5′-3′)
GAPDH	AACGACCCCTTCATTGACC	GACCACGACTCATACAGCAC
Nrf2	GCTCAACTTGCATTAATTCGGG	CTCTTTTCGGAAGTGGATGACT
HO-1	TCTTCACCTTCCCCAACATTG	GTACTGTGGTTCCTGGTCTC
NQO1	ATGGTCGGCAGAAGAGC	TGAAGTTAGGGTAGTAAAGG
GSTA2	TGAGGAACAAGATGCCAAGC	GGAATAAAGGTCGAAGGGAGAC
PPAR*α*	TCATCTCCTACTGTCCCACG	CAGGACCGGAAGATTTGCATC
CPT1	TCATCTCCTACTGTCCCACG	CATAAATACCGTCACCCTCGA
SCD	CCCTACGGCTCTTTCTGATC	GACAGTTTCTCTTCCCCTCATG
NF-*κ*B	CGAGCTTGTAGGAAAGGACTG	CTGATGCTGGACTTACGACAC
IL-1	GTACATCCTCGACGGCATC	GACCAGAAAACCTCAAACTCCA

**Table 2:** The content of TFC and TPC and antioxidant activity of EE, EAE, EF-B, and EF-D (IC_50_, *μ*g/mL).

Samples	IC_50_ (*μ*g/mL)		*A*_0.5_ (*μ*g/mL)	TFC (mg RE/g extract)	TPC (mg GAE/g extract)
	DPPH	ABTS	FRAP		
EE	101.715 ± 2.007	3.110 ± 0.493	37.972 ± 1.579	281.9 ± 2.0	195.536 ± 2.0
EAE	156.956 ± 2.370	7.071 ± 0.849	34.980 ± 1.544	335.3 ± 25.9	110.58 ± 4.77
EF-B	300.190 ± 2.477	5.915 ± 0.772	53.823 ± 1.731	106.4 ± 113.7	108.83 ± 2.08
EF-D	64.380 ± 1.809	5.531 ± 0.743	21.271 ± 1.328	397.1 ± 5.0	165.95 ± 1.45
VC	4.625 ± 0.665	2.613 ± 0.417	nd	nd	nd
BHT	nd	nd	75.688 ± 1.879	nd	nd

IC_50_: concentration that causes 50% scavenging in radical concentration; TFC: total flavonoid content; RE: rutin equivalents; TPC: total phenolic content; GAE: gallic acid equivalents; nd: not determined. All values are represented as mean ± SD (*n* = 3).

## Data Availability

The data used to support the findings of this study are included within the article.
